# Potential Therapeutic Strategies for Alzheimer's Disease Targeting or Beyond ***β***-Amyloid: Insights from Clinical Trials

**DOI:** 10.1155/2014/837157

**Published:** 2014-07-17

**Authors:** Qiutian Jia, Yulin Deng, Hong Qing

**Affiliations:** School of Life Science, Beijing Institute of Technology, 5 South Zhongguancun Street, Haidian District, Beijing 100081, China

## Abstract

Alzheimer's disease (AD) is a progressive neurodegenerative disorder with two hallmarks: *β*-amyloid plagues and neurofibrillary tangles. It is one of the most alarming illnesses to elderly people. No effective drugs and therapies have been developed, while mechanism-based explorations of therapeutic approaches have been intensively investigated. Outcomes of clinical trials suggested several pitfalls in the choice of biomarkers, development of drug candidates, and interaction of drug-targeted molecules; however, they also aroused concerns on the potential deficiency in our understanding of pathogenesis of AD, and ultimately stimulated the advent of novel drug targets tests. The anticipated increase of AD patients in next few decades makes development of better therapy an urgent issue. Here we attempt to summarize and compare putative therapeutic strategies that have completed clinical trials or are currently being tested from various perspectives to provide insights for treatments of Alzheimer's disease.

## 1. Introduction

Alzheimer's disease (AD) is named after the German physiologist who first presented neuropathological characteristics of the dementia at the 37th meeting of Society of Southwest German Psychiatrists in 1906 [1]. Alzheimer's studied a patient with progressive memory loss for five years and analyzed the brain post mortem using silver staining [2]. This contributed to the identification of neuritic plagues and neurofibrillary tangles (NFT) [1], two characteristics employed to identify the dementia to date. It took another 20 years to determine *β*-amyloid (A*β*) and tau which are major components of neuritic plagues and NFTs, respectively [3], marking the modern era of study of AD research.

AD, as a progressive neurodegenerative disorder, deprives patients of their memory and even lives. Memory loss is the most notable symptom [4, 5] at the early stage but as the disorder advances, difficulties with language, perception, and execution of movement become prominent [6], followed by neuropsychiatric and behavioral abnormality, muscle mass loss, and mobility deterioration [6]. Loss of normal daily living in those with dementia is inevitable.

In addition to the affliction and sufferings to patients, Alzheimer's disease can cost society substantially, especially in developed countries. The expenditure of AD was around $100 billion per year [7]; the bill was about €177 billion in Europe solely in 2008 [8]. Due to deteriorating abilities to live on their own, caregivers are necessary for progressed AD patients. Burdens on these caregivers' life including physical, psychological, and economic aspects [9–11] can be a major concern.

The global prevalence of dementia for people over the age of 60 is estimated as high as 40 million in 2001, and the figure is forecasted to double every 20 years [12, 13], indicating that Alzheimer's disease has become a modern epidemic. In the near future, surging number of AD patients will become an overbearing social issue. Therefore, the need for therapeutic strategies for this devastating disease is urgent.

Currently, Food and Drug Administration (FDA) approved AD drugs are still limited within two categories: cholinesterase inhibitors and memantine [14–16] (a NMDA receptor antagonist). Unfortunately, the effects and benefits of these drugs are marginal and work only to alleviate the symptoms [17–19]. However, in recent years, fundamental researches focusing on the pathogenesis of AD paved the way for development of new treatments targeting the radical source of Alzheimer's disease [20]. Numerous trials have been or are currently being conducted to determine effects of various compounds on AD in different stages.

Alzheimer's disease causes major impairment of individual health and social economy due to the limited effective therapeutic approaches. With the explosive explorations based on two hallmarks of AD, numerous clinical trials targeting on or off A*β* have been or are being conducted. In this paper, we will briefly summarize successes and failures in clinical trials in Alzheimer's disease and try to give a systematic review in an attempt to derive insights from previous experience.

## 2. Therapeutic Targets Focusing on A*β* Cascade Hypothesis (Table 1)

### 2.1. Inhibition of A*β* Production

**Figure 1:**
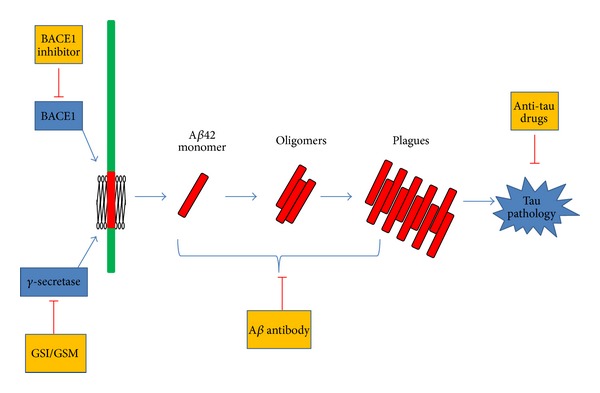
*β*-amyloid hypothesis based therapeutic targets. APP, after sequentially being cleaved by BACE1 and *γ*-secretase, gives rise to a neuron toxic molecule A*β*42. This peptide can exist as monomers or aggregates into oligomers and plagues. The assembly of A*β*42 triggers downstream effects and induces tau phosphorylation. BACE1 inhibitors and GSI/GSM aim to prohibit the production of pathological A*β*, and vaccines or A*β* antibodies promote clearance mechanism. As for tau, GSK-3*β* inhibitors and other antiaggregates are potential therapeutics targeting on blocking tau hyperphosphorylation or aggregation.

Studies of familial Alzheimer's disease (FAD) motivate the discovery of responsible genetic factors, establishing A*β*-centered theory for AD. Amyloid precursor protein (APP) experiences sequential cleavages by *β*-secretase and *γ*-secretase and gives rise to the dementia culprit *β* amyloid (A*β*) that is thought to initiate soluble oligomers, insoluble fibrils, and accumulated plagues (Figure 1). APP can be alternatively processed by *α*-secretase within the A*β* region and generate a longer C-terminal fragment under the first cleavage. In terms of curbing production of A*β*, the three crucial enzymes processing APP have been therapeutic targets in drug development. The rationale is to inhibit *β*-/*γ*-secretase while promoting the *α*-secretase activity to become the priority strategy.

#### 2.1.1. *β*-Secretase (BACE1) Inhibitor

Beta-site APP-cleaving enzyme 1 (BACE1) is the protease responsible for the initial cleavage of APP, giving rise to the production of neurotoxic suspect A*β* [21, 22]. Mounting evidence corroborate the availability of BACE1 inhibition. BACE1 knock-out mice indicated a close correlation between the BACE1 inhibition and the A*β* decline [23, 24]. It is reported that BACE1 inhibition improved memory deficits [25] and rescued A*β*-driven cholinergic dysfunction [26] in APP transgenic mice. Although the BACE1-deficient animal model presented a relatively benign phenotype with high viability, suggesting that the possibility of targeting *β*-secretase would be a safe therapeutic approach, further testing indicated that the drastic inhibition would result in hypomyelination and behavioral abnormalities such as seizures [27–30]. This is because, except from APP, BACE1 has a series of substrates, like neuregulin-1, related to myelination [29, 31]. AD pathology onset was postponed in the APP × BACE1+/− mice; however, it hinted at a partial inhibition that might mitigate the potential safety problems [32, 33]. It has been noted that the discrepancy between potency-required molecular weight and CNS penetration-required size [34, 35] poses another challenge.

Many BACE1 inhibitors are derived from approved drugs for type 2 diabetes with properties regulating insulin metabolism. Nuclear peroxisome proliferator activated receptor gamma (PPAR*γ*) functions as a transcription factor regulating gene expression [36], modulating inflammation response, promoting microglia-mediated A*β* endocytosis, and declining cytokine secretion [37]. Thiazolidinediones can activate PPAR*γ* to inhibit *β*-secretase and promote ubiquitination to degrade amyloid load [38]. PPAR*γ* agonists like thiazolidinediones derivatives rosiglitazone and pioglitazone soften the peripheral insulin resistance [39], which aggravates AD neuropathology, and this decline of insulin sensitivity helps in A*β* proteolysis. The study of rosiglitazone has been developed to a large phase 3 trial; however, it has been discontinued due to cardiac risk concerns [40]. Pioglitazone has recently progressed into a phase 3 clinical trial after precluding a previously reported bladder risk. But due to the involvement of substrate complexity and some adverse effects, other phase 3 clinical trials for BACE1 inhibitors are still lacking.

However, several novel drugs are currently under investigation. Based on conjugation to a penetrant carrier peptide [41, 42], the potent CNS impermeable compound, CTS-21166, has completed the phase 1 trial. It showed a good tolerance and a reduction of plasma A*β* level in healthy volunteers [43]. A phase 1b dose-escalating study for MK-8931 demonstrated a positive effect in reducing the level of toxic proteins in addition to safety and good tolerance. A phase 2 trial recruiting 200 mild-to-moderate patients was expanded to a larger 1960-participant phase 3 trial, including conventional cognitive and functional primary outcomes, and it recently passed an interim safety evaluation.

Another BACE1 inhibitor, LY2886721, though it appeared to be safe and lowered A*β*42 in cerebrospinal fluid by more than two-thirds in phase 1 trial [44], was terminated due to the fact that 4 out of 45 patients showed liver abnormalities during the phase 2 trial. Besides, RG-7129 was also terminated in its phase 3 trials in 2013. These terminations again signaled that significant challenges are remaining: whether BACE1 inhibitors will be safe in the long run and if lowering BACE1 activity will slow cognitive decline.

#### 2.1.2. *γ*-Secretase Inhibitors (GSI) and Modulators (GSM)

*γ*-secretase is a transmembrane protease responsible for the eventual cleavage of amyloid precursor protein (APP) to generate A*β* (Figure 1), thus it is considered a principal therapeutic target in Alzheimer's disease [45, 46]. This enzyme complex consists of four components: Aph1, Pen2, glycosylated nicastrin, and endoproteolyzed presenilin as the catalytic core [47], and it is involved in myriads of physiological process. The versatility places hurdles in the way of *γ*-secretase targeted drug development. In the human body, aside from APP, there are more than 50 different substrates that *γ*-secretase is capable of reacting with, many of which are neuronal substrates [48]. Importantly, *γ*-secretase is also responsible for cleavage of Notch 1, which leads to the release of the Notch intracellular domain (NICD), subsequently translocated to the nucleus to regulate genes involved in cell development, cell survival, and cell fate determination [49]. Thus, inhibition of *γ*-secretase needs to be cautiously designed to particularly circumvent the drawbacks caused by Notch signaling abnormality. Haematological [50] and gastrointestinal [51] toxicity, skin reactions [52, 53], and changes to hair color [54] are the most commonly reported adverse effects of *γ*-secretase inhibitor.

Several *γ*-secretase inhibitors (GSIs) have been launched in clinical trials. Many reduced the A*β* production in plasma or CSF (cerebrospinal fluid), but few successfully avoided the Notch-induced side-effects. Semagacestat decreases A*β* level in plasma and downregulates its generation in the central nervous system (CNS) [55]. Semagacestat is the first *γ*-secretase inhibitor that have been taken into Phase 3 clinical trials. While phase 1 trial suggested a dose-dependent decline of A*β* synthesis in CSF [55], phase 2 trial began exhibiting skin-related side effects. Although A*β* level in plasma has significantly decreased, it was not duplicated in CSF and no effects on cognition and function were found. Two pivotal phase 3 trials were reluctantly started; however they were discontinued due to increased risk of skin cancer and infection and lack of efficacy [56]. Fall of semagacestat, a potentially promising drug candidate, repeated disappointing results of other GSIs, which deemed that a deeper understanding of interaction between 4 subunits and their substrates is necessary.

Different GSIs present favor to interact with subunits of *γ*-secretase, exhibiting target specificity. DAPT and L685458 indicated the smallest selectivity, while MRK-560 and sulfonamide based GSIs strongly prefer to inhibit PS1 instead of PS2 [57, 58]. Aph1 heterogeneity is critical for individual survival, suggesting that targeting of Aph1b *γ*-secretase specifically would be more tolerated [59], although the feasibility of drug design still remains difficult to determine.

Accordingly, the second generation Notch-sparing *γ*-secretase inhibitors aimed at selective inhibition of specific sites took the spotlight. Avagacestat (BMS-708163), begacestat, and NIC5-15 are such Notch-sparing GSIs under clinical trials. It was reported that avagacestat (BMS-708163) has 137-fold selectivity for APP over Notch in cell culture and robustly reduces CSF A*β* levels without causing Notch-related toxicity in rats and dogs, although this is still being researched [60]. Phase 2 trials have to be terminated due to the adverse effects of gastrointestinal and dermatological system in addition to the lack of cognitive improvement compared to placebo counterparts. Begacestat decreased the A*β* concentration in the plasma but not in CSF [49, 61], and a phase 1 clinical trial in combination with cholinesterase inhibitor donepezil has been completed, further data was unavailable. Another Notch sparing GSI candidate, NIC5-15, a natural monosaccharide [62], is currently under a phase 2 trial and demonstrated good tolerance and safety [63].

Given that the unresolved adverse effects brought on by GSIs are tricky to address, the concept of *γ*-secretase modulators (GSMs) was established with the expectation of nonsteroidal anti-inflammatory drugs (NSAIDs). A subset of NSAIDs, like ibuprofen, indomethacin, and sulindac sulfide, disconnected from their cyclooxygenase (COX) properties were discovered to be able to selectively reduce the production of A*β*42 at the cost of elevated shorter peptide A*β*38 [64, 65]. Surprisingly, this downregulation of toxic A*β* level lacks the inhibitory effect on Notch or other substrates [64]. This finding promoted the GSMs as promising therapeutic candidates for Alzheimer's disease, because the Notch-induced drawbacks may be avoided and on the other hand, the overproduction of shorter and more soluble A*β*38 seems less likely to aggregate and is less pathogenic.

Among the compounds described above, tarenflurbil (R-flurbiprofen) relates to ibuprofen structurally and pharmacologically. Phase 1 trials with a broad dose range (400 to 1600 mg/day) revealed a low drug exposure in the brain [66], while phase 2 trials narrowed this spectrum (400–800 mg, twice daily) and showed trivial benefits on function with the lowest dosage (400 mg). Although undesirable side effects like nausea, dizziness, and diarrhea were observed, this compound is still considered tolerable [67]. After modification, phase 3 trial suggested neither functional improvement nor clinical efficacy in the mild AD patients [68] and thus the results were disappointing. The weak potency of tarenflurbil can be attributable to low CNS penetration as shown in phase 1 trial, and on the other hand, NSAID residue activity curbed A*β* clearance mechanism mediated by microglia [69].

Another GSM CHF-5074 based on R-flurbiprofen ameliorated brain A*β* load and improved the animals' performance in behavior tests. The drug's safety and tolerability have been evaluated and are undergoing a phase 2 trial. Published data indicated that this compound may have an additional function of acting independently of A*β*42 [70, 71]. Nevertheless, a balance between lipophilicity and potency of these compounds must be considered. The remarkably increased potency in 2nd and 3rd generations of GSMs relies heavily on the increase of lipophilicity, which has been proved to result in off-targets, like hepatotoxicity [72].

#### 2.1.3. *α*-Secretase Activator

APP can be cleaved by an alternative *α*-secretase rather than *β*-secretase in the first step to circumvent the generation of pathological A*β* peptide. Hence, increasing the chance of *α*-cleavage could be an effective approach to decrease the A*β* formation and promote soluble APP production to protect neurons [73]. Agonists of muscarinic, glutamate, and serotonin receptors (and the agonists or antagonists of transmitters receptors would be discussed in following section), statins, oestrogens, testosterone, and protein kinase C activators belong to this drug classification that can motivate *α*-secretase activity, and they have been launched in clinical trials, but data indicating their use in AD is limited [74].

Etazolate (EHT-0202), a selective GABA_A_ [75] receptor modulator, has completed a phase 2 trial in patients with mild to moderate AD. It presented a good oral bioavailability and an elevation of sAPP*α* [76]. Bryostatin-1, a macrocyclic lactone, caused a decline of brain A*β*40/42, improved behavior test in AD mouse model [77], and was under a phase 2 trial, but the specific information is inaccessible.

Statin drugs such as atorvastatin and simvastatin lower peripheral cholesterol production to prevent heart attacks and other expressions of cardiovascular disease. Atorvastatin, in combination with cholinesterase inhibitor, has completed a phase 2 clinical trial and achieved a beneficial cognition and function [78], but failed to repeat the outcome in a 641-patient phase 3 clinical trial [79, 80]. Simvastatin can penetrate BBB and long-term statin treatment can decline A*β* level. In a 35-normal participant phase 4 trial of Simvastatin, it was reported to reduce phospho-tau-181 in CSF, while not total tau or A*β* level [81]. A follow-up study evaluating one year simvastatin treatment in 120 cognitively normal and middle-aged adults, effect on CSF levels of A*β*42, t-tau, and p-tau181, is ongoing.

### 2.2. Anti-*β*-Amyloid Aggregation

The pathological A*β* peptides, prone to assembly into aggregate as neuro-/synaptic toxic products spur the idea of inhibition of A*β* aggregation or destabilization of the A*β* oligomers species. However, A*β* aggregations are characterized with a high stability resistance to disaggregation [82] and remain insoluble even with heat or SDS [83]. The fact that amyloid fibrils have an extremely low energy state [82] and the lack of thorough understanding of A*β* aggregation process have complicated the issue. Besides, another challenge would be to access the compounds with high CNS bioavailability and low immunogenicity and toxicity. It is generally believed there are three strategies that block A*β* aggregation: antiaggregate compounds, metal complexing agents and immunization. They can disturb the formation of either soluble oligomers or insoluble plagues.

#### 2.2.1. Nonpeptidic Antiaggregates

The first class of mentioned inhibiting aggregation compounds is nonpeptidic antiaggregates, tramiprosate, derived from proprionic acid that is a primitive representative. The promising outcomes of this agent from the safety and tolerance [84] were neutralized by two following phase 3 trials: the European trial precluded methodological problems that might lead to the negative in the North American trial and demonstrated the poor CNS penetration and the weak potency [85] of this drug.

The second generation of nonpeptidic antiaggregates was expected to meet those challenges. Scyllo-inositol is thought to effectively impede A*β* aggregation, promote misfolding modulation, and accelerate aggregates disassociation [86]. Because this compound can cross blood brain barrier (BBB), with the assistance of inositol transporters, it can achieve a high concentration in CNS via peripheral administration. This drug is being tested in the phase 2 trial with mild-to-moderate Alzheimer's disease patients on the basis of good tolerance and safety profile [87]. Although high doses (1000 mg and 2000 mg) resulted in serious adverse effects, the studies continued to test the low dose (250 mg) cohorts [88]. Epigallocatechin-3-gallate (EGCg), a polyphenol from green tea, via disrupting unfolded peptide, stimulated *α*-secretase activity and inhibited A*β* aggregation in animal models [89]. This agent was also involved in modulation of cell transduction, regulation of cell survival and death [89], and protection of mitochondrial function. The multiple effects of this natural compound make it a promising candidate, and a phase 3 trial with early AD patients with EGCg is being conducted.

#### 2.2.2. Metal Complexing Agents

After A*β* peptides were produced and released into extracellular fluids, metals like Zn and Cu can motivate oligomerization into fibrils. So metal chelators or metal complexing agents that can interfere with reaction of metal ions with A*β* are likely to be a therapeutic strategy. Clioquinol (PBT2), metal-induced A*β* inhibitors, also has a potent CNS permeability. PBT2 can redistribute metal ions to neurons promoting metalloproteinase expression and thus an increment of A*β* degradation. A phase 2 trial was completed and it proved a decrease of A*β*42 concentration in CSF and an improvement of cognitive and behavioral performance [90].

#### 2.2.3. Active Immunization

It is conventionally thought that clearance of CNS A*β* requires a BBB permeability property, confining the therapeutic targets in a very narrow realm: medicinal chemistry-driven and small molecules. Nonetheless, incredible work done by Schenk et al. revealed that immunization of PDAPP transgenic mice markedly mitigated amyloid plaque burden, improved neuritic dystrophy, and even reduced existed A*β* plagues [91]. This striking breakthrough suggested that A*β* immunotherapy would be a potential strategy to remove both soluble and aggregated *β* amyloid [92].

AN-1792, the first anti-A*β* vaccine (with full length A*β* 1–42) tested in active immunization clinical trial, was terminated in the phase 2 trial in patients with early AD due to the fact that some participants developed aseptic meningoencephalitis and cerebral microhemorrhage [93]. The complication is attributed to cytotoxic T cell or autoimmune response [94–96]. Therefore, employing only fragments instead of full length of A*β* or other cell epitopes to circumvent toxicity and inflammation is highlighted. Additionally, the security of adjuvant and delivery approaches must be cautiously considered.

The next generation of vaccine is devoid of any T-cell epitopes. CAD-106, consisting A*β* 1–6 peptides coupled to a Q*β* virus-like particle, has recently completed the phase 2 trial in patients with mild AD and did not lead to meningoencephalitis [97]. Two other vaccines, UB 311 (A*β* 1–14) and V950 (A*β* N-terminal conjugated to ISCO-MATRIX), both containing B-cell epitopes, have also recently finished phase 1 trial. However, another vaccine AC-001(A*β* 1–7 conjugated to inactivated diphtheria toxin) discontinued its phase 2 trial in August 2013, because the studied drug elicited a strong antibody response. Another active immunization approach is developed on the foundation of Affitope using short six-amino acid peptides that imitate the native A*β* sequence. AD-01 and AD-02, targeting N-terminal fragments of A*β*, were proved to rescue AD-like symptoms in animal models [98]. Recently, AD-02 has progressed into a phase 2 clinical trial.

#### 2.2.4. Passive Immunization

Another strategy to avoid immune response is direct administration of antibodies. This passive immunization has an approximate potency to remove amyloid plaques and rescue neuritic and glial pathology [99], reduce early tau hyperphosphorylation [100] and cytopathology [101], and reverse abnormal hippocampus synaptic plasticity [102].

Bapineuzumab (AAB-001) is a humanized monoclonal antibody, derived from 3D6, published to promote removal of A*β* plagues and rescue synapse loss in APP transgenic mice brain [99]. However, in a 234-patient phase 2A safety and tolerability trial, this agent indicated no significant alteration on primary measures of cognition and daily activity. For apolipoprotein E (ApoE) *ε*4 carriers, there is a temporary vasogenic oedema, an adverse effect correlated with dose administration [87]. Given that 4,000 mild AD patients across North America and Europe showed no treatment effect on either cognitive or functional outcomes, the phase 3 trial was terminated. Solanezumab (LY2062430) is a humanized monoclonal IgG1 antibody directed against the mid-domain of the A*β* peptide (A*β* 16–24) and designed to specifically bind soluble species of A*β*. Phase 2 study showed dose-dependent increases of various A*β* species in plasma and CSF, an indication that insoluble A*β* is released from plagues and leaches into fluid [103]. Two trials in phase 3 suggested a limited benefit for cognitive performance as compared to cholinesterase-inhibitor drugs. A third trial started from July 2013 to test demonstrated brain amyloid burden, and data is expected to be read out in December 2016. Gantenerumab, a human IgG1 antibody binding to A*β* fibrils, can elicit phagocytosis to remove A*β* plagues in brain and rescue A*β* oligomers that induced impaired long-term potentiation (LTP) in rats model. An expanded phase 2/3 trial of 770 participants is being conducted and is estimated to be completed in 2016. Crenezumab, a novel humanized antibody with IgG4 backbone, is believed to limit microglia mediated inflammatory cytokines release to avoid vasogenic oedema. It can recognize *β* amyloid oligomers, fibrils, and plagues with a high binding affinity. Phase 1 study confirmed safety and tolerance, followed by an ongoing phase 2 trial in patients with mild to moderate AD using elevated dose as well as a test for prevention of this progressive dementia. There are several antibodies which have completed or have undergone the early clinical trials, as shown in Table 1.

Many monoclonal antibodies are delivered intravenously, whereas passive immunization can be also accomplished via infusion of intravenous of immunoglobulins (IVIg) from healthy donor. A small study in 8 patients showed increase of A*β* antibodies in serum, decrease of A*β* in CSF, and stabilization of MMSE (mini-mental-state exam) scores over 18 months. A phase 2 trial with 24 patients suggested beneficial cytokine concentrations alteration in plasma. However, two critical phase 3 trials showed no difference between study drug and placebo, though a trend toward benefit for the higher dose, and thus were halted. Another published phase 2/3 clinical trial evaluating infusion of albumin in combination of IVIg is currently conducted in patients with mild to moderate AD.

Active immunization maintains the body with a constant high concentration of immunoglobulin, so this strategy calls for fewer follow-up injections with a reasonable expense. But to tackle with the T-cell induced inflammation would be a tricky issue. Passive immunization is a more effective method especially for elderly people considering their weakened responsiveness to vaccines [104]. Selection of safe epitopes can be readily met, as well as a better control of antibody titer; however, antibody delivery could be inconvenient and costly, and the risk of vasogenic oedema and cerebral amyloid angiopathy might increase.

### 2.3. Tau

According to A*β* hypothesis, intracellular neurofibrillar tangles (NFTs) induced by altered phosphatase/kinase activity is a downstream event of aggregation of *β*-amyloid (Figure 1), and NFTs as a catalyst will aggravate the oxidation and further result in neuronal dysfunction, cell death, and transmitter deficits. Tau is normally a highly soluble protein in cytoplasm binding to microtubules as a stabilizer. Formation of NFTs as a result of hyperphosphorylated and misfolded tau protein aggregation is toxic to neurons. The pathological tau proteins lose the capability to aid microtubules in transporting neuronal substance, leading to neuronal dysfunction and apoptosis [105, 106].

#### 2.3.1. Kinase Inhibitors

Protein kinase, a group of critical enzymes responsible for tau overphosphorylation, is a prerequisite for the tau-induced toxicity. However, myriads of kinases mutually play a central role in regulating cell function and guaranteeing a normal physiological condition. The development of tau-targeted therapy is therefore challenging due to redundancy of kinase interactions and uncertainty of which enzyme specifically catalyzes the phosphorylation that we are focusing on [107, 108].

The first class of tau inhibitors aims to modulate tau phosphorylation via decreasing the activity of related kinase since imbalanced interaction between glycogen synthase kinase 3 beta (GSK3*β*) and protein phosphate 2 (PP2A) enhances tau hyperphosphorylation and NFT formation [109]. GSK3*β* appears to engage in AD pathogenesis given its impact on cellular signaling and gene description [109]. Recently, it has been reported that GSK3*β* is responsible for 31% of the pathological phosphorylation sites of tau protein [110] and is found colocalized with NFTs in postmortem brain [111, 112]. Toxic A*β* that promotes GSK3*β* activity bridges a link between the two hallmarks of Alzheimer's disease [110], implicating that GSK3*β* inhibitor is a potential drug target.

Lithium and valproate reduced tau phosphorylation and prevented reversed aspects of tauopathy in animal models [113] but did not show cognitive improvement in clinical trials with AD patients [114]. NP-031112 (NP-12), a non-ATP competitive inhibitor of GSK3*β*, counteracts tau phosphorylation, reverses amyloid burden in brain, prevents cell loss, and rescues spatial memory deficits using animal models [115]. But the phase 2b trial was terminated due to the negative results. Development of some paullone, indirubin, and maleimide family-derived GSK3*β* inhibitors is in the pipeline, yet stuck in the preclinical trials concerning the cytotoxic effects.

Cyclin dependent kinase 5 (cdk5) is another kinase tightly associated with tau pathology. Cdk5 regulating protein was found in AD brain and thus is probably causing a pathophysiological tau phosphorylation [116]. Cdk5-selective inhibitors were demonstrated to penetrate BBB and reduce elevated A*β* level by regulating cdk5 [117] and are at preclinical status. The test of several compounds targeting other protein kinases, like cdk1/2/9, p38, Erk1/2, JNK, casein kinase, and DYRKIA brought disappointing outcomes, and trials were discontinued due to the poor efficacy or severe adverse effects.

#### 2.3.2. Inhibition of Tau Aggregation

Another scenario to interfere with tau-induced NFT is to inhibit tau aggregation or promote tau assembly disassociation. Rember (methylene blue) is such a tau antiaggregant [118]. Preclinical data revealed a learning deficit reversing property and a completed phase 2 trial proved that this agent can slow down AD progression with a good bioavailability [119, 120]. TRx0237, another methylene blue, has an improved drug absorption, bioavailability, and tolerability. Since 2008, intensive investigation of this agent began, and growing evidence indicated that TRx0237 benefits neuroprotection [121] and A*β* clearance in transgenic mice and improves spatial learning in rats [119, 122]. The antiaggregation properties were reported by some papers, and three phase 3 studies are ongoing.

Epothilone D (BMS-241027) is a microtubule stabilizer, via inhibition of tau release from microtubule to maintain the transportation function of axon, and on the other hand, precludes formation of tau aggregation. This agent restored behavioral and cognitive deficits, inhibited neuron loss, and curbed the tauopathy in animal models [123, 124]. Epothilone can penetrate BBB and exert a better efficacy at low concentration and now undergoes a phase 1 clinical trial. Nicotinamide, the precursor of coenzyme NAD+, reduces phosphorylated tau and protects microtubules stabilization in mouse model [125]. Nicotinamide has been launched into clinical studies suggesting that it is safe and well tolerated and a phase 2 clinical trial is ongoing in patients with mild-to-moderate Alzheimer's disease.

## 3. Putative Therapies Still Derived from Neurotransmitter System

Neurotransmitters depletion (basically referring to acetylcholine, ACh) and synaptic dysfunction are two classical features of AD [126]. Thus, two hypotheses have been established—cholinergic hypothesis [127] and glutamatergic hypothesis [128], based on which FDA approved therapies—AchE inhibitors and NMDA receptor antagonists—to mitigate AD symptoms were developed. Although drugs regulating transmitters' production, release, and recycling cannot prevent the progression of AD, pursuit of searching novel receptor agonists and antagonists has never stopped (Table 2).

Cholinergic neurons impairment accompanies the early progression of dementia. From animal and human studies, cholinesterase inhibitors administration stimulated memory and learning process [129]. Besides, a marked correlation between loss of cholinergic neurons and deterioration of defected memory was proved in animal models later [130, 131]. Therefore, improvement of cholinergic system, including potentiating effects of acetylcholine (Ach) and inhibiting activity of cholinesterase, is a potential therapeutic goal.

Ach is a ligand for nicotine receptors and exerts an excitatory effect on the postsynaptic neuron, an essential event for long-term potentiation (LTP) and memory formation. Several nicotinic receptor agonists to reinforce this event are being tested in clinical trials. EVP-6124, a selective agonist of the *α*-7 nicotinic acetylcholine receptor, has finished a phase 1/2 trial showing safe and well tolerated results and recently (Oct 2013) entered two phase 3 trials to test the cognitive benefits. Quite a few other clinical trials testing nicotinic agonists are ongoing (ladostigil hemitartrate, phase 2; ispronicline, phase 1), completed (RO5313534), or terminated (ABT-089).

A transmitter that indirectly modulates neuron degeneration and memory deficits is serotonin (5-HT). Growing evidence indicated that inhibition of 5-HT_6_ could facilitate Ach release and via elevated cholinergic transmission, memory and learning defects were likely to be ameliorated. 5-HT_6_ antagonists were widely reported in many studies to rescue anticholinergic drugs-induced amnesia [132]. Recently, two agents, PRX-03140(5-HT_4_ antagonist) and SB-742457(5-HT_6_ antagonist), completed the phase 2 trials. Lu AE58054, an antagonist of the serotonin 6 (5-HT_6_) receptor was recently progressed into a phase 3 trial with 930 mild to moderate AD patients in combination with AchE inhibitor donepezil.

## 4. Potential Findings of Therapeutics for Alzheimer's Disease from Other Perspectives

In addition to the two hallmarks and neurotransmitter system impairment, there are several other features found in Alzheimer's disease, including inflammation, oxidative stress, mitochondrial dysfunction, neurotrophin deficiency, and so forth. These aspects are not systematically and thoroughly summarized and are likely to be neglected though; they do provide new perspectives in developing AD treatments. Many drugs of great therapeutic potential are under clinical trials (Table 3).

### 4.1. Anti-Inflammation and Antioxidants

Chronic inflammation is an essential feature of AD and contributes to its pathogenesis in numerous ways. Microglia are brain's resident macrophages that monitor brain activity and play a contributing role in removal of redundant and apoptotic neurons [133, 134], remodeling of normal synapse [135], and protection of CNS from pathogens and detritus [136]. However, they can shift to another phenotype to secrete series of inflammatory factors, exerting detrimental effects on bystander neurons and processes they are involved in. Aggregated A*β* appears to be a robust agent driving this alteration, since markers of activated microglia were densely colocalized within the deposits [137, 138]. Microglia seem incapable of degrading A*β* that they intake [139, 140], leading to a frustrated phagocytosis instead. As clinical trials have been a major disappointment, agents that drive microglia to a phenotype that favors attack on pathogens rather than bystander neurons may hold therapeutic potential.

Based on compelling evidence of the involvement of inflammation in AD pathogenesis, anti-inflammatory drugs have been investigated. COX inhibitors, aiming to reverse the elevated A*β* burden and cognitive deficits caused by overexpression of COX2 [141, 142], showed limited efficacy [143]. Glucocorticoid steroids, considered as potent drugs by declining overexpression of proinflammatory mediators [144], showed poor benefits [145] or adverse effects [146]. Flavonoid administration prevented cognitive impairment associated with inflammation in animal studies [147, 148]; however, the beneficial effects cannot be repeated in human [149].

Another anti-inflammatory agent etanercept, an approved arthritis drug, is a TNF-*α* antagonist to neutralize the activated microglia secreted cytokines. Modulation of immune system may have benefits for Alzheimer's disease patients and a phase1 clinical trial in combination with supplementation of some specific nutrients is ongoing in mild to moderate AD patients. Curcumin, a natural polyphenol, has anti-inflammatory and antioxidant properties and exhibits other neuroprotective functions like promoting metal chelation, curbing tau aggregation, and facilitating neurogenesis. It undergoes a phase 2 study, but details are not available.

Oxidative injury is the following causal event of inflammation and the study of antioxidants in treatment of AD achieved little success. Alpha-tocopherol, a synthetic vitamin E, is thought to prevent brain cell damage by destroying toxic free radicals and slowing down the cognitive decline in the finished phase 3 trial. In addition, a phase 3 trial of DHA (docosahexaenoic acid), an omega-3 fatty acid, was terminated because cognitive decline was not changed compared to placebo group.

### 4.2. Mitochondrial Dysfunction

Mitochondrial dysfunction taking place in early AD enhances synaptic damages and neuron apoptosis, so it is considered a causal factor of neurodegeneration [150]. APP and A*β* are transported into mitochondrion reacting with mitochondrial components, leading to an impaired ATP processing and increased oxidative stress level [150, 151]. ApoE4, a risk factor for sporadic AD, harms mitochondrial trafficking and function and promotes mitochondrial apoptosis [152, 153]. Replacing mitochondrial DNA (mtDNA) form one cell line with mtDNA from AD patients supported a mitochondrion cascade hypothesis [154], offering new therapeutic targets. Latrepirdine (dimebon), an antihistamine that preserves mitochondrial structure and function and protects against A*β* induced pore apoptosis, has been tested in a clinical trial in Russia and phase 2 data showed improvement of all outcomes [155] while phase 3 trial did not confirm it [156]. However, a combination of therapy with donepezil was demonstrated as well tolerated from preliminary results in phase 1 trial and further information awaits analysis [157]. AC-1204 is designed to improve mitochondrial metabolism [158] by induction of chronic ketosis, thus rescuing regional cerebral hypometabolism presented in early Alzheimer's disease, and this agent is undergoing a phase 3 clinical.

### 4.3. Diabetes

Diabetes is another risk factor for Alzheimer's disease [159] in which the insulin resistance and disrupted glucose metabolism [160] can be attributed to a tumor necrosis factor (TNF) induced inflammation pathway [161, 162]. Insulin can mediate A*β* degradation by activating insulin-degrading enzyme (IDE) [163]. A CSF insulin decline in prodromal female AD patients [164], the presence of insulin resistance, and the dysfunctional insulin signaling pathway in dementia brain [165] are documented. Incretin and liraglutide, two drugs for hyperglycemia, implicating beneficial effects on AD mice [166, 167], reinforced the relationship between diabetes and AD, and a phase 2 study of liraglutide, a glycogen like peptide 1 agonist is still ongoing. These evidences brought the advent of concept “type 3 diabetes,” [168] and an intranasal insulin delivery with an ameliorating cognitive function effect [169] has completed its phase 2 study.

### 4.4. ApoE (Apolipoprotein) and A*β* Export

ApoE (apolipoprotein) is a powerful genetic factor [170, 171] for sporadic AD beyond APP, PS1, and PS2 genes. The isoform ApoE4 substantially promotes the risk of AD and decreases the age of onset [172]. ApoE is generally thought to regulate A*β* clearance and thus influence fibrillogenesis. In CNS, ApoE, responsible for transportation of cholesterol to neurons, is primarily produced in astrocytes [173]. A*β* aggregation and clearance are differently affected in an isoform (*ε*2, *ε*3, and *ε*4) dependent manner; frequency of AD and mean age at clinical onset are 91% and 68 years of age in *ε*4 homozygote, 47% and 76 years of age in *ε*4 heterozygote, and 20% and 84 years in *ε*4 noncarriers [172, 174]. ApoE was found colocalized with amyloid plagues [175] and this coexistence is more abundant in ApoE4 carriers [176]. Additionally, ApoE4 is associated with cognition decline before clinically apparent syndromes [177, 178]. ApoE4, as previously described, can work synergically with other risk factors, like insulin resistance and peripheral vascular diseases [179, 180], thus exerts a confounding effect on AD and triggers inflammatory cascade. After being synthesized, ApoE is lipidated by the ABCA1, a process regulated by nuclear receptor liver X receptor (LXR) or retinoid X receptor (RXR), and transported to form lipoprotein particles. The complex particle binds soluble A*β*, promoting transfer via neuron surface receptors such as low-density lipoprotein receptor (LDLR), low-density lipoprotein receptor-related protein 1 (LRP1), and heparin sulphate proteoglycan (HSPG) [181, 182] into neurons where degradation can be finished with proteolysis in lysosome. ApoE *ε*4 isoform has less affinity of binding A*β* compared to *ε*3, showing a less efficient clearance phenotype [183, 184]. Stimulation of LXR/RXR enhances removal of A*β* [185, 186] while inhibition of ABCA1 impairs A*β* clearance in ApoE4 rather than ApoE3 mice [187]. Therefore, the molecules and receptors involved in ApoE metabolism can be potential therapeutic targets for drug development.

Recent studies demonstrated that oral administration of bexarotene, a RXR agonist and a FDA approved anticancer drug, reduces A*β* plaques and improves cognitive function in an ApoE-dependent manner in amyloid mouse model [186], and a phase 2 clinical trial is currently ongoing to determine its safety and effect on abnormal proteins in the brain with 300 mg for one month compared to placebo. Other drugs that aim to regulate ApoE expression (LXR agonist TO901317) [185, 188], block ApoE-A*β* interaction, disrupt ApoE4 domain (CB9032258, phthalazinone analogue) [189], mimic the receptor binding region [190] (COG112), and so forth, have shown benefits of reversing A*β* burden in vivo or in vitro, but did not reach the clinical trials yet. ApoE-targeted therapies are still at the early stage of development and relevant approaches and strategies are required to carefully evaluate them though, showing a huge promising battle with Alzheimer's disease.

### 4.5. Neurotrophin

Nerve growth factor (NGF) as a neurotrophin plays a critical role promoting survival and maintaining the function of cholinergic neurons [191, 192]. In AD patients, transcription and translation levels of NGF were changed [193, 194], suggesting that NGF supplementation probably is a treatment approach for Alzheimer's disease. NGF with unfavorable size and polarity is a peptide that cannot cross BBB [193, 195], so to safely and efficiently deliver it to the brain will be a great challenge [196, 197]. However, efforts have been made to overcome this obstacle. An example of strategy is as follows: CERE-110 uses adeno-associated virus to transfer a gene that makes NGF and is injected into AD patients' brain. This approach undergoes a phase 2 study.

## 5. Concluding Remarks

A*β* cascade hypothesis was firstly proposed in 1992 [198] assuming that *β*-amyloid would be the suspect initiating pathogenesis of Alzheimer's disease. So a series of explorations focusing on physiological and pathological processes that participate in the production, aggregation, and clearance of A*β* have been widely studied. The identification of two crucial enzymes (*γ*-secretase and BACE1), responsible for the cleavage of the presumably pathogenic A*β* from its precursor, suggests that the cure of AD may be around the corner.

However, failures in many large clinical trials using A*β*-targeted drugs (Table 4) and FDA approved compounds with marginal efficacy questioned the validity of A*β* cascade hypothesis. Indeed, A*β* hypothesis, having dominated the AD realm for two decades, has always been controversial. One of the most unfavorable evidences was the finding that amyloid plagues were diffused in AD patients' brain postmortem (and neuroimaging outcomes confirmed the autopsy findings), which is abundant in healthy people [199, 200]. Nevertheless, plenty subsequent investigations put forward the oligomeric form of A*β*, rather than plagues, as the actual culprit for synapse dysfunction [201, 202] and the following amplifying events. This significant finding, at least partially, defended the validity of A*β* cascade hypothesis. But, still, why do therapeutic strategies targeting the secretases only have marginal efficacy? First, the two versatile secretases (BACE1 and *γ*-secretase) are at the same time responsible for processing other substrates, which unfortunately are either vital to metabolism normality or tricky to avoid targeting. The undesirable side effects are so overwhelming that they prohibit drug's efficacy and approval. Second, the drug permeability through blood brain barrier (BBB) is another considerable problem. Most drugs described above have a poor capability to cross BBB, so it is reasonable to see numerous clinical trials, including those having progressed to phase 3, fail. Instead of questioning the plausible hypothesis, it is more imperative to cautiously design clinical studies and interpret the outcomes.

Given limited benefits from inhibition of A*β* production, more focus should be converted to the clearance strategy. Delivery of antibodies may be a good choice due to the safety leverage compared to vaccine. Besides, there are quite a few ongoing clinical trials using passive immunization. From Table 1, antibodies are capable of binding and clearing multiple forms of A*β*. It is important because there is equilibrium between oligomers and plagues of A*β* [203]. For a single-target antiaggregate disrupting formation or enhancing disassembly of A*β* oligomers, plagues as a reservoir will replenish and maintain the balance [204, 205]. So the property of simultaneously interfering different processes during A*β* aggregation suggested that passive immunization might be of a promising value.

In recent years other AD risk factors have been widely studied. Though no groundbreaking outcomes have been shown, it provided quite a few unprecedented opportunities. First, the validated AD specific biomarkers need to be carefully developed and examined. Biomarkers should be able to at least precisely indicate the response to therapeutic intervention to avoid misinterpretation of clinical trial data. Besides, current animal models have serious limitations. Most transgenic mouse models published in AD studies overproduce A*β* solely mimicking familial Alzheimer's disease, might not suffice phenotypes of sporadic AD accounting for the dominant populations.

In addition, AD is a disorder that is too intricate and too factor-driven to be entirely understood from its pathogenesis. As we discussed previously, various factors (A*β*, tau, inflammation, and apoE) complicatedly interact with each other. So the conventional “one protein, one drug, one disease” hypothesis would not work for Alzheimer's disease. From the successful experience in therapeutic development in multifactorial diseases like AIDS, atherosclerosis, cancer, and depression, multitarget drugs or combination therapy can possibly generate more benefits. Since drugs with more than one target could possibly mitigate a redundancy effect in such a complex nerve network, this combination therapy or similar approach multitarget-directed ligands (MTDLs) might bring new hope in search of therapeutics for Alzheimer's diseases [206, 207]. In this novel fashion, some combinations with approved drug are under clinical trials (Tables 1, 2, and 3, RCTs marked with ∗).

Notwithstanding these challenges, with more scientific insights from basic researches and cooperation between laboratories and pharmaceutical companies, it is very likely to find the optimum treatment for Alzheimer's disease in the near future.

## Figures and Tables

**Table 1:** RCTs based on β-amyloid in recent years.

Mechanism	RCT	Status	Estimated end	Dementia stage	Enrollment	Duration	Reported outcomes	Details of drugs/RCTs
↓Aβ production								
BACE1 inhibitor	Pioglitazone	Phase 2; completed	2005.1	Mild-to-moderate	25	18 months		Insulin sensitizer, class of PPARγ agonists
	CTS-21166	Phase 1; completed	2008.2	Healthy	56			
	MK8931	Phase 3; ongoing	2018.3	Mild-to-moderate	1960	≈6.5 years		With enhanced BBB permeability
	E2609	Phase 1; completed	2013.9	MCI/mild AD	65			
GSI/GSM	NIC5-15	Phase 2; ongoing	2013.12		40			Notch-sparing, insulin-sensitizer
	Begacestat	Phase 1; completed	2009.10	Elder healthy	49		Dose-dependent changes in plasma Aβ levels	Selectively inhibits cleavage of APP over Notch [208]
	CHF 5074	Phase 2; completed	2012.4	MCI	96	12 weeks		NSAID
	EVP-0962	Phase 2; completed	2013.10	MCI/early stage	52	14 days		
α-secretase activator	Atorvastatin	Phase 3; completed∗	2007.7	Mild-to-moderate	600	80 weeks		Tested with AchEI
	Simvastatin	Phase 3; completed	2007.10	Mild-to-moderate	400	18 months		
	Etazolate	Phase 2a; completed	2009.8	Mild-to-moderate	159		Safe and well tolerated	↑α-secretase activity, acting as a GABA-A receptor modulator and a PDE-4 inhibitor [209]
	Epigallocatechin-3-gallate (EGCg)	Phase 2/3; ongoing	2015.6	Early stage	50	18 months		Prevents the Aβ aggregation via binding to the unfolded peptide
↓Aβ aggregation/oligomers	Scyllo-inositol (ELND005/AZD103)	Phase 2; completed	2010.5	Mild-to-moderate	350	18 months	Insufficient to support/refute benefits [210]	
	Tramiprosate (3APS)	Phase 3	unknown	Mild-to-moderate	950		Suggesting disease-modifying effects [211]	
	PBT2	Phase 2; completed	2007.12	Mild AD	80	12 weeks	Well-tolerated, ↓CSF Aβ42, and improved executive function [212]	

↑A*β* clearance								
Active immunotherapy	Affitope AD02	Phase 2; completed	2013.12	Early stage	335	>1 year		N-terminal Aβ1-6, a synthetic peptide
	Affitope AD03	Phase 1; completed	2011.11	Mild-to-moderate	28			i.h. with or without adjuvant aluminum
	UB 311	Phase 1; completed	2011.4	Mild-to-moderate	19			N-terminal Aβ1-14
	V 950	Phase 1; completed	2012.1		86			formulated on Aluminum-containing adjuvant
	CAD 106	Phase 2; completed	2012.12	Mild AD	177		A favourable safety profile [213]	N-terminal Aβ1-6; i.m. of adjuvanted CAD106;
Passive immunotherapy	BAN2401	Phase 2; ongoing	2016.12	MCI/mild AD	800	18 months		mAb against Aβ oligomers
	BIIB037	Phase 1; ongoing	2014.11	Prodromal to mild	160			Administered via intravenous (IV) infusions in subjects
	Ponezumab	Phase 2; completed	2011.8	Mild-to-moderate	198	24 months		
	Crenezumab	Phase 2/3; ongoing	2016.5	Mild-to-moderate	361	24 months		
	Gammagard (IVIg)	Phase 2, completed	2010.4	Mild-to-moderate	24	6 months	Improved cognition	
		Phase 3; completed	2012.12	Mild-to-moderate	390	70 weeks	Showed no significant effect	
		Phase 2; ongoing	2014.10	MCI	50	24 months		
	AMBAR	Phase 2/3; ongoing	2016.12	Mild-to-moderate	350			
	Gantenerumab	Phase 3; ongoing	2019.3	Mild	1000	>5 months	Phase 1 RCT↓brain Aβ; high doses, AE	Mainly targets Aβ plagues
	Solanezumab	Phase 3; ongoing	2016.12	Mild	2100		No benefits in primary outcomes	Mainly targets soluble oligomeric Aβ
	AAB-003	Phase 1; ongoing	2014.8	Mild-to-moderate	104	52 weeks		Previously treated with AAB-003
	GSK933776	Phase 1; completed	2011.5		50			
	SAR228810	Phase 1; ongoing	2015.1	Mild-to-moderate	48	14.5–22 months		

Anti-tau								
↓tau production	Valproate	Phase 3; complete	2009.12	Mild-to-moderate	313	2 years	Did not show cognitive benefits and prevention of behavioral defects; associated with reduced brain volumes	
	Lithium	Phase 2; ongoing	2019.4		80		A pilot study was insufficient to support or refute the efficacy [214]	
↓tau fibrillization/deposition	Nicotinamide	Phase 1/2; ongoing	2014.7	Mild-to-moderate	50	24 weeks		Vitamin B3
	TRx0237	Phase 3; ongoing	2015.12	Mild/mild-to-moderate	700/833	18 months/15 months		
	Methylene blue (Rember)	Phase 2; completed		Mild-to-moderate	321	6 months	Showed uncertain results	
	Davunetide (AL108)	Phase 2; completed	2008.1	MCI	144	12 weeks	Showed benefits on memory	
	BMS-241027	Phase 1; completed	2013.10	Mild	40	9 weeks		

RCT: randomized controlled trial; PPAR: peroxisome proliferators activated receptor; BBB: blood brain barrier; MCI: mild cognitive impairment; GSI: *γ* secretase inhibitor; GSM: *γ* secretase modulator; NSAID: nonsteroidal anti-inflammatory drugs; AChEI: acetylcholinesterase inhibitor; GABA: *γ*-aminobutyric acid; PDE: phosphodiesterase; CSF: cerebrospinal fluid; i.h.: subcutaneous injection; i.m.: intramuscular injection; mAb: monoclonal antibody; AE: adverse event.

∗RCTs with a combination of another drug.

Data sources: http://www.clinicaltrials.gov/.

**Table 2:** RCTs targeting neurotransmitter systems in recent years.

Mechanism	RCT	Status	Estimated end	Dementia stage	Enrollment	Duration	Reported outcomes	Details of drugs/RCTS
Cholinergic agents								
AchE inhibitor	Huperzine A	Phase 2/3; completed	2012.6	Mild-to-moderate	390	6 months	↑cognitive function, daily living activity, global clinical assessment	A natural AChEI; antioxidant and neuroprotective properties [215]
	Ladostigil hemitartrate	Phase 2; ongoing	2016.9	MCI	200	36 months		Antioxidant properties; modulates APP processing
Nicotinic receptor agonist	EVP-6124	Phase 3; ongoing	2017.7	Mild-to-moderate	790	26 weeks	Positive outcomes in a 24-week phase 2b RCT	
	RO5313534	Phase 2; completed	2010.11	Mild-to-moderate	389	6 months		*α*7 nicotinic receptor agonist; as add-on therapy to donepezil
	Ispronicline (AZD3480)	Phase 2; ongoing	2014.7	Mild-to-moderate	300	1 year		*α*4*β*2 and *α*2*β*2 nicotinic receptor agonist
	MT-4666	Phase 2; ongoing	2015.5	Mild-to-moderate	450	24 weeks		
	ABT-089	Phase 2; terminated	2013.10	Mild-to-moderate	434	24 weeks		
	MK-7622	Phase 2b; ongoing	2017.8	Mild-to-moderate	830	12–24 weeks	As adjunctive therapy to donepezil	*α*7 receptor modulator

Glutamatergic agents	AVP-923	Phase 2; ongoing	2014.9	Mild-to-moderate	200	10 weeks	Behavioral problems	NMDA receptor antagonist

Serotoninergic agents	Lu AE58054	Phase 3; ongoing∗	2016.1	Mild-to-moderate	≈2500		Positive results in a phase 2 RCT, 278 participants, 6 months;	Several phase3 RCTS with donepezil (AchEI);
	SB-742457	Phase 2; completed	2011.8	Mild-to-moderate	684	6 months	showed positive results	

RCT: randomized controlled trial; AChEI: acetylcholinesterase inhibitor; MCI: mild cognitive impairment; NMDA: N-methyl-D-aspartic acid.

Data sources: http://www.clinicaltrials.gov/.

∗RCTs with a combination of another drug.

**Table 3:** Other novel approaches in AD clinical trials.

Mechanism	RCT	Status	Estimated end	Dementia stage	Enrollment	Duration	Reported outcomes	Details of drugs/RCTS
Anti-inflammation and antioxidation	Curcumin	Phase 2; completed	2007.12	Mild-to-moderate	33			NSAID, cholesterol-lowering properties
	Etanercept	Phase 1; ongoing	2015.6	Mild-to-moderate	12	12 months	↑cognitive function with other nutrients.	Approved drug for arthritis; may modulate immune system; benefit AD patients
	dl-alpha-tocopherol (vitamin E)	Phase 3; completed	2012.10	Mild-to-moderate	613			
	PUFA	Phase 1/2; ongoing∗	2015.1		100	18 months		Tested alone or together with lipoic acid
	RO4602522	Phase 1; completed	2013.5		17			

PDE inhibitors	PF-04447943	Phase 2, completed	2010.9	Mild-to-moderate	198			Selective PDE 9A inhibitor
	MK0952	Phase 2; completed	2007.11	Mild-to-moderate				Selective PDE 4 inhibitor
	Cilostazol	Phase 4; completed	2013.7	Mild-to-moderate	46			PDE3 inhibitor, Antiplatelet agent in WMHI; ↑pCREB

Tyrosine kinase inhibitor	Masitinib	Phase 3; ongoing∗	2015.12	Mild-to-moderate	396			In combination with AChEI and/or memantine

Insulin and GLP1-R agonists	Intranasal insulin (glulisine)	Phase 2/3; ongoing	2015.2	MCI/mild AD	240	12 months		
	Exendin-4 (exenatide)	Phase 2; ongoing	2016.7	MCI/early stage	100	3 years	Showed neuroprotection	Diabetes agent
	Liraglutide	Phase 2; ongoing	2017.1	early stage	206	12 months		

Modulating mitochondrial function	AC-1204	Phase 2/3; ongoing	2015.1	Mild-to-moderate	480	26 weeks		
	Latrepirdine (Dimebon)	Phase 3; completed	2009.12	Mild-to-moderate	598	6 months		

RXR agonist	Bexarotene	Phase 2; ongoing	2014.3	Mild-to-moderate	20			Approved anticancer drug; linked to key pathways relevant to AD and A*β*

NGF delivery	CERE-110	Phase 2; ongoing	2014.12	Mild-to-moderate	50	24 months	24 months	Designed to help neurons function better; uses a virus to transfer NGF gene
	Encapsulated Cell biodelivery of NGF	Phase 1b	2011.12		6	12 months		

RCT: randomized controlled trial; NSAID: nonsteroidal anti-inflammatory drugs; PDE: phosphodiesterase; WMHI: subcortical vascular disease; pCREB: phosphorylated cAMP-response element binding protein; AChEI: acetylcholinesterase inhibitor; GLP1-R: glucagon-likepeptide1 receptor; MCI: mild cognitive impairment; RXR: retinoid X receptors; NGF: nerve growth factor.

∗RCTs with a combination of another drug.

Data sources: http://www.clinicaltrials.gov/.

**Table 4:** Terminated trials targeting A*β* hypothesis.

Mechanism	RCT	End	Enrollment	Duration	Main reasons
BACE1 inhibitor	Rosiglitazone	2009.2	693	24 weeks	Unimproved cognitive status
	LY2886721	2013.8	128		AE: 4 cases of liver damage in a phase 2 study in June 2013

GSI/GSM	Semagacestat	2011.5	164	>7 months	Unimproved cognitive status, but worsening functional ability; AE: skin cancers and infections
	Avagacestat	2010.6	209	24 weeks	AE: gastrointestinal and dermatological abnormalities like diarrhea, nausea, vomiting, rash, and itching skin; nonmelanoma skin cancers; and worsened cognition
	tarenflurbil	2008.5	1684	18 months	Insufficient pharmacodynamics: poor capability to penetrate the BBB

Active immunology	AN1792	2003.9	375		AE: 6 patients developed aseptic meningoencephalitis due cytotoxic T cell response in phase 2a trial
	ACC-001	2014.2	126	24 months	Showed a serious side effect in phase 2 trial

Passive immunology	Bapineuzumab (AAB-001)	2012.6	1331	18 months	Showed no treatment effect on either cognitive or functional outcomes in two phase 3 trials

GSK3*β* inhibitor	Tideglusib (NP12)	2012.6	306	45 weeks	Missed its primary endpoint and some secondary endpoints

RCT: randomized controlled trial; AE: adverse event; BBB: blood brain barrier.

Data sources: http://www.clinicaltrials.gov/; http://www.alzforum.org/.

## Abbreviation

AD: Alzheimer's disease
A*β*: Amyloid *β*
NFT: Intracellular neurofibrillar tangles
FDA: Food and Drug Administration
APP: Amyloid precursor protein
NMDA: N-Methyl-D-aspartic acid
PS: Presenilin
NICD: Notch intracellular domain
GSI: *γ*-Secretase inhibitors
CSF: Cerebrospinal fluid
CNS: Central nervous system
CSF: Cerebrospinal fluid
GSM: *γ*-Secretase modulators
NSAID: Nonsteroidal anti-inflammatory drugs
BACE1: Beta-site APP-cleaving enzyme 1
FAD: Familial Alzheimer's disease
BBB: Blood brain barrier
COX: Cyclooxygenase
GABA: *γ*-Aminobutyric acid
ApoE: Apolipoprotein E
MMSE: Mini-mental-state exam
GSK3*β*: Glycogen synthase kinase 3 beta
PP2A: Protein phosphatase 2
cdk5: Cyclin dependent kinase 5
TNF: Tumor necrosis factor
IDE: Insulin-degrading enzyme
LXR: Liver X receptor
RXR: Retinoid X Receptor
LDLR: Low-density lipoprotein receptor
LRP1: Low-density lipoprotein receptor-related protein 1
HSPG: Low-density lipoprotein receptor-related protein 1
ABCA1: ATP-binding cassette transporter 1
Ach: Acetylcholine
LTP: Long-term potentiation
5-HT: Serotonin
NGF: Nerve growth factor.
